# Transcriptomic signatures of cellular and humoral immune responses in older adults after seasonal influenza vaccination identified by data-driven clustering

**DOI:** 10.1038/s41598-017-17735-x

**Published:** 2018-01-15

**Authors:** Emily A. Voigt, Diane E. Grill, Michael T. Zimmermann, Whitney L. Simon, Inna G. Ovsyannikova, Richard B. Kennedy, Gregory A. Poland

**Affiliations:** 1 Mayo Clinic Vaccine Research Group, Mayo Clinic, Rochester, MN 55905 USA; 2 Division of Biomedical Statistics and Informatics Mayo Clinic, Rochester, MN 55905 USA

## Abstract

PBMC transcriptomes after influenza vaccination contain valuable information about factors affecting vaccine responses. However, distilling meaningful knowledge out of these complex datasets is often difficult and requires advanced data mining algorithms. We investigated the use of the data-driven Weighted Gene Correlation Network Analysis (WGCNA) gene clustering method to identify vaccine response-related genes in PBMC transcriptomic datasets collected from 138 healthy older adults (ages 50–74) before and after 2010–2011 seasonal trivalent influenza vaccination. WGCNA separated the 14,197 gene dataset into 15 gene clusters based on observed gene expression patterns across subjects. Eight clusters were strongly enriched for genes involved in specific immune cell types and processes, including B cells, T cells, monocytes, platelets, NK cells, cytotoxic T cells, and antiviral signaling. Examination of gene cluster membership identified signatures of cellular and humoral responses to seasonal influenza vaccination, as well as pre-existing cellular immunity. The results of this study illustrate the utility of this publically available analysis methodology and highlight genes previously associated with influenza vaccine responses (e.g., CAMK4, CD19), genes with functions not previously identified in vaccine responses (e.g., SPON2, MATK, CST7), and previously uncharacterized genes (e.g. CORO1C, C8orf83) likely related to influenza vaccine-induced immunity due to their expression patterns.

## Introduction

Worldwide, influenza affects 5–10% of adults annually, and results in an estimated 250,000 to 500,000 deaths^1^. Influenza morbidity and influenza-associated deaths increase significantly with age^2,3^, and more than 90% of influenza-associated deaths occur in individuals ≥65 years of age^4^. Although seasonal influenza vaccination offers protection against severe influenza disease, levels of protection vary between seasons, individuals, and age—tending to be lower in elderly populations^5–12^. In fact, the effectiveness of seasonal trivalent inactivated influenza vaccination among community-dwelling older adults has been estimated to be only 30–40%^6,12–14^. With the aging of populations in the U.S. and globally, it is imperative that influenza vaccine-induced immunity in older adults be better understood^15–17^.

Systems vaccinology and vaccinomics, the application of systems biology to the study of vaccines, are a promising method to better understand human immune responses to vaccines from a holistic perspective^18,19^. A seminal paper by Querec *et al*. in 2008 applied a systems biology approach to study yellow fever vaccine-induced immunity and identified novel genes involved in vaccine-induced antibody and CD8+ T cell responses whose expression levels could predict immunogenicity of the vaccine in subjects, setting a new standard for vaccine studies^20^. Such systems biology approaches provide complementary insights to reductionist approaches by revealing novel interactions between immune system processes critical to developing immune responses to vaccines^21^. Systems biology approaches have been previously applied to the study of seasonal influenza vaccination in humans, resulting in the identification of important players in, and predictors of, immune responses. These include the frequencies of specific immune cell subsets, pre-existing immunity from previous influenza vaccination or infection, and gene expression signatures of immune response in *ex vivo* human immune cells^9,22–28^.

Such systems studies of the human response to vaccination require complex analytical methods to mine important immune-related information out of large datasets. In particular, the in-depth study of transcriptional changes in peripheral blood mononuclear cells (PBMCs) post-vaccination may lead to a better understanding of the development of humoral and cellular immune responses after influenza vaccination; however, systems-level characterization of PBMC responses to vaccination requires analytical techniques to prune large transcriptomics datasets to the subset of biologically relevant genes. As transcriptomic datasets are large (thousands of genes at multiple time points), the identification of single genes as predictors of immune responses is challenging^29,30^. This issue is exacerbated in biological situations where marginal associations are weak and/or noisy, leading to high rates of false-positive identifications. PBMCs also represent a complex mixture of immune cell types, each with its own changing pattern of gene expression, inherently providing additional complexity to transcriptomic datasets. As genes work within networks, not individually, effective analytical methods to identify important drivers of immunity that focus on groups of genes may better model the mechanisms of response^31^.

Weighted Gene Correlation Network Analysis (WGCNA) is a new data-driven clustering algorithm that can be used to identify clusters of genes that act similarly across individuals^32,33^. This gene clustering method was developed in order to effectively study transcriptomic data from complex systems, such as human disease states and plant microbiome interactions^32,34–36^. For biological scenarios where with low signal-to-noise ratios and weak marginal associations, WGCNA cluster analysis has been demonstrated to be more reproducible and less prone to finding false positives than marginal meta-analysis statistical techniques^37^. WGCNA has been utilized to identify subsets of genes from transcriptomic datasets that are involved in the biological questions studied, while excluding genes that are likely unrelated^38–40^. To date, WGCNA has been sparsely used in the study of human immunology, and thus further validation of this technique for such applications, and comparisons of results to those of previous systems studies of influenza vaccination in humans is essential. We tested the utility of WGCNA in analyzing transcriptomic profiles of PBMCs from older adults after seasonal influenza vaccination. The algorithm generated fifteen gene expression clusters, eight of which were highly enriched for immunity-related genes. These immune-relevant clusters had distinct and biologically interpretable functions, and cluster gene expression correlated with subject immune responses corresponding to those biological functions. These gene clusters allowed us to identify independent marker genes for the development of cellular (PBMC cytokine secretion) and humoral (serum antibody, B-cell ELISPOT) immunity. These results compared well with previous studies using alternative analysis approaches. Further study of these clusters identified likely involvement of specific immune cell subsets in the development of cellular, memory B-cell, and antibody immune responses.

## Materials and Methods

The study population and laboratory methods described herein are similar or identical to those published in our previous studies^41–46^.

### Recruitment

**Figure 1:**
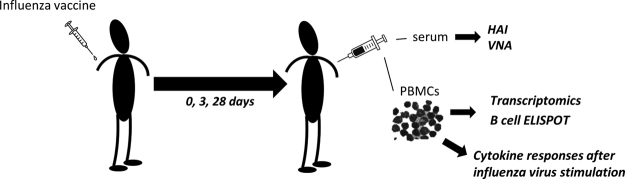
Study design. Adults were vaccinated with trivalent seasonal influenza vaccine. At Days 0, 3, and 28 post-vaccination, blood samples were drawn and serum antibody (HAI, VNA) titers measured. PBMCs were assayed for anti-influenza memory activity by influenza A/H1N1-specific B-cell, and mRNA transcriptomic profiles measured by NextGen sequencing. PBMCs were also stimulated *in vitro* by influenza A/H1N1 virus, and resulting cytokine secretion (IFNγ, IL1β, IL-2, IL-4, IL-6, IL-8, IL-10, IL-12p70, IL-13, TNFα) was measured by ELISA.

A cohort of generally healthy recipients of the 2010/11 seasonal trivalent inactivated influenza vaccine (TIV; Fluarix; containing the A/California/7/2009 NYMC X-191 (H1N1), A/Victoria/210/2009 NYMC X-187 (H3N2; an A/Perth/16/2009-like virus), and B/Brisbane/60/2008 viral strains) was used for this study^41,47^. Subjects were 50–74 years of age, and vaccination was administered by standard protocol into the deltoid muscle using a 1 inch needle. Vaccine lot #AFLUA524AA (GlaxoSmithKline) was used for all subjects. Blood samples (100 ml each) were collected before vaccination (Day 0) and at Days 3 and 28 post-vaccination (Fig. 1)^41^. Recruitment was performed at Mayo Clinic, Rochester, MN; a complete dataset was obtained from 138 individuals, and these datasets were used for further analysis.

### Ethics Statement

Written informed consent was obtained from each participant, and the Mayo Clinic Institutional Review Board approved the study. Review Board approved the study. All methods were performed in accordance with the relevant guidelines and regulations.

The virus was propagated in nine-day-old embryonated chicken eggs obtained from Charles River Laboratories in Storrs, CT, USA. Mayo Clinic’s Institutional Animal Care and Use Committee (IACUC) policy does not require review of research on unhatched embryonated eggs destroyed before hatching, in accordance with the Office of Laboratory Animal Welfare and the National Institutes of Health policy.

MDCK cells were obtained from the American Type Culture Collection.

### Isolation of peripheral blood mononuclear cells

PBMCs were isolated from whole blood samples as described previously using BD Vacutainer® CPT_TM_ Cell Preparation Tubes with sodium citrate^43^. The cells were then resuspended in freezing medium, aliquoted, and cryopreserved for future use^42–44^.

### Growth of influenza virus

The influenza A/California/7/2009/H1N1-like virus strain used in this study was provided by the Centers for Disease Control and Prevention (Atlanta, GA). As previously described, the virus was propagated in embryonated chicken eggs and harvested from the allantoic fluid. 50% tissue culture infectious dose (TCID_50_) measurement of virus stock titers was conducted by infection of MDCK cells with serial dilutions of the virus and addition of red blood cells (RBC) five days after infection. Hemagglutination (HA) was then determined in wells with successful virus replication^42,44,48–50^

### Previous measurement of immune response to influenza vaccine

Hemagglutination-inhibition influenza A/H1N1 antibody (HAI) titers, virus-neutralizing influenza A /H1N1antibody (VNA) titers, and influenza A/H1N1-specific B-cell ELISPOT counts for these subjects before and after vaccination were previously published by our group^42,44–46^. Participants’ influenza A/H1N1-specific HAI titers were measured from sera at each timepoint pre- and post-vaccination. Serum influenza A/H1N1-specific neutralizing antibody titers were measured by a cell-based microneutralization assay at each timepoint with influenza A/H1N1 virus stimulation (200 plaque-forming units per 5 µl), as previously described^46^. Quantification of influenza A/H1N1-specific B cells (memory-like IgG B cells) was performed on subjects’ PBMCs using the MabTech Human IgG ELISpot^PLUS^ Kit (Mabtech, Inc.; Cincinnati, OH), as described previously^44^. Influenza A/H1N1 virus stock was diluted 1:50 (50,000 TCID_50_/well) before coating the ELISPOT plates.

### *In vitro* chemokine/cytokine assays

PBMCs were thawed, counted, and plated at 2 × 10^5^ cells per well in 96 well plates. Six wells were plated per subject; three of these were stimulated with influenza A/California/7/2009/H1N1-like virus (MOI 0.5) and three left unstimulated. Two additional wells were stimulated with 5 μg PHA (positive control). Plates were incubated for 48 hours at 37 °C, after which supernatants were harvested, plated in a new 96-well plate, and frozen until use. The Meso Scale Diagnostics (MSD; Rockville, MD) V-PLEX Proinflammatory Panel 1 (human) Kit was used according to the manufacturer’s protocol to measure 10 cytokines (IFN-ɣ, IL-10, IL-12p70, IL-13, IL-1β, IL-2, IL-4, IL-6, IL-8, and TNF-α) in the aforementioned supernatants. Cytokines were categorized as T_H_1 (IFN-γ, IL-2), T_H_2 (IL-10, IL-4), and pro-inflammatory (TNFα, IL-6); the data for each cytokine were transformed to a uniform scale and then averaged within-category.

## Statistical Methods

### RNA-seq

The mRNA sequencing methods were identical to those published in previous transcriptomics studies^51^. Briefly, total RNA was extracted from each cryopreserved subject PBMC sample using RNeasy Plus mini kits and RNAprotect reagent (Qiagen; Valencia, CA). cDNA libraries were created in Mayo Clinic’s Gene Sequencing Facility using the mRNA-Seq. 8 Sample Prep Kit (Illumina; San Diego, CA), and poly-A RNA was isolated using magnetic purification. Single-end read sequencing was performed using an Illumina HiSeq. 2000. Sequencing reads were aligned to the human genome build 37.1 using TopHat (1.3.3) and Bowtie (0.12.7). Quality control and normalization of the mRNA-sequencing gene counts data are as described by Ovsyannikova *et al*.^51^ Briefly, gene counts were normalized using Conditional Quantile Normalization^52^, and 14,197 genes with at least 32 counts at one of our three timepoints (Day 0, 3, or 28) were used in subsequent analyses.

### WGCNA

Weighted Gene Coexpression Network Analysis (WGCNA) was used to create data-driven phenotype-agnostic gene clusters using the Day 28 normalized gene expression data^33,53,54^. For all pairs of genes, a co-expression similarity matrix was calculated as Sij = |0.5 + 0.5*cor(x_i_, x_j_)|. This signed similarity matrix was used to construct an adjacency matrix, calculated as A_ij_ = S_ij_^β^, with soft-thresholding to preserve the strength of the correlation between the genes. The parameter β was selected as the smallest value that achieves scale free topology (β = 12 for this analysis). The topological overlap dissimilarity was calculated from the adjacency matrix, and hierarchical clustering was used to define the gene clusters. Each gene cluster’s eigengene is used as a summary of the cluster’s gene expression activity, representing the first principal component of the gene expression levels within the cluster. Pearson’s correlation was used to correlate subject immune phenotypes with each cluster’s eigengene to identify which clusters were related to vaccination responses.

### Gene enrichment analyses

We performed enrichment analysis using the RITAN^55^ package (https://github.com/MTZimmer/RITAN) and leveraging previously published Blood Translation Modules (BTMs)^56^.

### Cluster-cluster interaction analysis

Databases of known regulatory interactions were examined using RITAN to further understand the biologic relationships between the individual genes found in the WGCNA gene clusters. Interactions from the Pathway Interaction Database^57^, Transcription Factor Encyclopedia^58^, a directed protein interaction network^59^, HPRD^60^, CCSB^61^, HumanNet^62^ (minimum score ≥ 0.0), and STRING^63^(minimum score ≥700) were accessed using RITAN. This integrated network resource provided a comprehensive summary of known interactions across the human proteome.

Using this interaction network, we computed a gene cluster interaction metric quantifying the extent to which the genes in two clusters are directly regulated by one another. The fraction of genes in one gene cluster that interacted with genes in the second gene cluster was calculated, then compared to the expected interaction of two randomly-generated gene clusters of the same size, as follows: for gene clusters *s*^1^ = {*g*_1_^1^, *g*_2_^1^,*…*, *g*_*n*_^1^} *and s*^2^ = {*g*_1_^2^, *g*_2_^2^,*…*, *g*_*m*_^2^} that contain ||*s*^1^|| = *n* and ||*s*^2^|| = *m* genes, let the neighbors of a gene be *A*(*g*). Further, let the neighbors of genes in cluster *s*^1^ that are also members of cluster *s*^2^ be denoted *A*(*s*^1^)^2^ and *U* the cluster union. Finally, let the overlap between the two clusters be: *O*(*s*^1^, *s*^2^) = *U*(*A*(*s*^1^)^2^, *A*(*s*^2^)^1^). The interactivity is formally defined as: *G* = ||*O*(*s*^1^, *s*^2^)||*/*||*U*(*s*^1^, *s*^2^)||. We used permutation tests to judge the significance of observed interactivity scores. To assess the significance of an individual cluster-cluster interactivity score, randomly selected groups of genes of the same respective sizes as the clusters were generated and interactivity was calculated. This was repeated 1,000 times, resulting in a randomly generated distribution of interactivity scores. We declared the original cluster-cluster interactivity value as significant when it was at least three median-based standard deviations away from the median of this empirical null distribution (p-value <0.001).

## Results

### Vaccination boosts cytokine responses of human PBMCs

**Figure 2:**
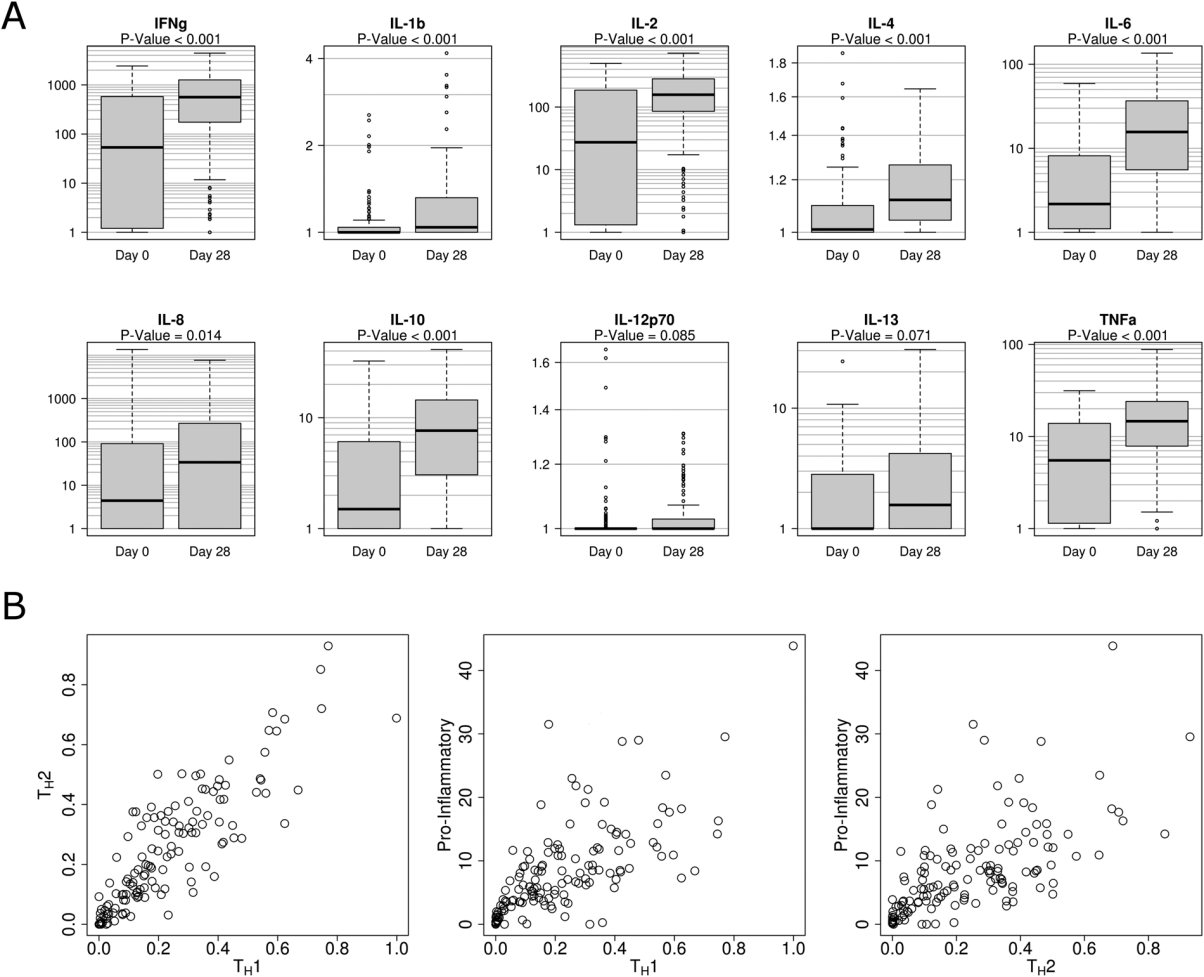
Influenza vaccination primes human PBMCs to secrete cytokines in response to influenza A/H1N1 virus stimulation. Panel A: Influenza A/H1N1-stimulated cytokine responses in PBMCs harvested prior to seasonal influenza vaccination were measurable, representing significant pre-existing immunity. However, cellular cytokine responses were significantly boosted above pre-vaccination levels for T_H_1 (IFN-γ, IL-2), T_H_2 (IL-4, IL-10), and proinflammatory (IL-6, TNF-α) cytokines. IL-1β, IL-12p70, and IL-13 cytokines were secreted at low levels approaching the assay limit of detection. IL-8 showed high levels of assay variability. Panel B: T_H_1, T_H_2, and pro-inflammatory post-vaccination (Day 28) cytokine responses are highly correlated.

In order to assess PBMC cytokine recall responses in response to influenza vaccination, we sampled subject PBMCs immediately prior to vaccination and 28 days post-vaccination. Harvested PBMCs from 138 subjects were stimulated *in vitro* with A/H1N1 influenza virus, and supernatants were removed after 48 hours and assayed for secreted cytokines (Fig. 2).

PBMCs collected post-vaccination produced significantly more IFN-γ, IL-1β, IL-2, IL-4, IL-6, IL-10, and TNFα in response to *in vitro* influenza A/H1N1 virus stimulation than PBMCs harvested prior to vaccination and identically stimulated (Fig. 2, panel A). Some individuals demonstrated high pre-vaccination PBMC cytokine recall responses, which is evidence of pre-existing immunity to influenza A/H1N1. Levels of IL-1β, IL-12p70, and IL-13 were deemed too low for meaningful analysis (i.e., post-stimulation samples below the assay limit of detection in 31%, 80%, and 20% of subjects, respectively). IL-8 responses were highly variable between replicates (standard deviation between replicates was more than triple that of other cytokines), and these data were accordingly removed from further analyses.

T_H_1 cytokines (IFN-γ, IL-2) were more highly secreted than pro-inflammatory cytokines (IL-6, TNFα), which are in turn more highly secreted than T_H_2 cytokines (IL-4, IL-10). Secreted cytokine levels in individuals are highly inter-correlated, with subjects typically showing consistently high (or low) secretion across T_H_1, T_H_2, and pro-inflammatory cytokines (Fig. 2, panel B). Secreted cytokines were not found to correlate significantly with VNA, HAI, or B-cell ELISPOT responses (data not shown).

### WGCNA clusters transcriptomic data from human PBMCs after vaccination according to expression patterns across subject

Gene expression (mRNA-Seq) data was previously collected from study subjects’ PBMC samples collected before and after vaccination^51,64^. This effort successfully measured expression levels of 14,197 genes across subjects before, three days after, and 28 days after vaccination. Full transcriptomic and immune datasets were finally available after strict quality control for 138 study subjects.

In order to identify genes that correlate highly with immune responses and are influential in vaccine-induced immunity, we clustered gene-expression data into groups of similarly behaving genes across our subjects using WGCNA^33^. This data-driven clustering method used average-linkage hierarchical clustering to group genes according to their expression patterns across the subjects. WGCNA clustering of Day 28 gene-expression levels, based on the expression across the 138 subjects with full transcriptomic and immune datasets, resulted in the construction of 14 non-overlapping gene clusters (Supplementary Table S1). The size of these gene clusters ranged from 54 genes to 2,790 genes. For simplicity, we assigned a color to each gene cluster and refer to each specific cluster by color coding throughout this work. There were 1,112 genes that did not fit into other clusters and were grouped into a fifteenth “grey” cluster.

### WGNCA gene clusters are highly enriched for specific immune cell types and/or immune-related functions

To investigate the biologic relevance of the gene clusters created by WGCNA, gene enrichment analysis was conducted on the 100 genes in each cluster with the highest correlation with the cluster eigengene, using Blood Translation Modules (BTMs) as references. BTMs were developed from a systems-biology study of responses to five human vaccines and represent many of the known gene signatures of vaccine-related immunological processes and immune cell types^56^. Results of the enrichment analysis are shown in Table 1. Similar enrichment results were obtained using the DAVID functional annotation and Gene Ontology analysis (data not shown)^65–67^.

**Table 1:** WGCNA-determined gene clusters are strongly enriched for genes linked to particular cell types and functions. The top 100 genes in each gene cluster most concordant with the cluster eigengene were tested for enrichment for BTMs. The top 3 enriched-for BTMs are listed for each cluster.

Cluster name	# genes	Enriched for: (Top 3 BTMs)	Strength of enrichment (p-value)	Summary class
Tan	115	mitotic cell cycle (M4.7)	<10^−25^	Cell cycle
		cell cycle and transcription (M4.0)	<10^−25^	
		cell cycle (I) (M4.1)	<10^−25^	
Greenyellow	135	plasma cells & B cells, immunoglobulins (M156.0)	<10^−25^	B cell activity
		enriched in B cells (I), (II), (VI) (M47.0, M47.1, M69)	<10^−25^	
		B cell surface signature (S2)	10^−23.3^	
		enriched in B cells (III)	10^−13.2^	
Yellow	1780	enriched in monocytes (II) (M11.0)	<10^−25^	Monocytes and inflammation
		cell cycle and transcription (M4.0)	<10^−25^	
		Monocyte surface signature (S4)	10^−11.7^	
		TLR and inflammatory signaling (M16)/lysosome (M209)	10^−10.5^	
Magenta	269	Platelet activation - actin binding (M196)	10^−22.3^	Platelets and monocytes
		platelet activation and blood coagulation (M199)	10^−14.5^	
		enriched in myeloid cells and monocytes (M81)	10^−14.9^	
Salmon	96	NK cells surface signature (S1)	10^−21.6^	NK cell activity
		enriched in NK cells (I) (M7.2)	10^−17.2^	
		enriched in NK cells (II) (M61.0)	10^−9.9^	
Purple	225	enriched in NK cells (I) (M7.2)	<10^−25^	NK and T cell activity
		enriched in NK cells (II) (M61.0)	10^−5.8^	
		enriched in T cells (I) (M7.0)	10^−5.8^	
Pink	289	immune activation - generic cluster (M37.0)	10^−6.7^	Monocyte activation
		enriched in monocytes (II) (M11.0)	10^−6.6^	
		CCR1, y and cell signaling (M59)	10^−4.9^	
Green	1144	enriched in T cells (I) (M7.0)	10^−5.6^	T cell activation
		T cell activation (I) (M7.1)	10^−4.7^	
		T cell activation (III) (M7.4)	10^−2.8^	
Black	629	T cell activation (I) (M7.1)	10^−4.5^	T cell activation
		enriched in T cells (I) (M7.0)	10^−4.4^	
		T cell activation (III) (M7.4)/T cell differentiation (M14)	10^−2.6^	
Blue	2504	TBA (M177.0)	10^−2.5^	none
		No further enrichment found	—	
Brown	1988	TBA (M177.0)	10^–0.9^	none
		No further enrichment found	—	
Grey	1112	No enrichment found	—	none
Turquoise	2790	No enrichment found	—	none
Red	1067	No enrichment found	—	none
Cyan	54	No enrichment found	—	none

The WGCNA algorithm, acting solely on transcriptomic data, was able to separate clusters of genes with similar transcriptomic patterns across the 138 subjects (i.e., high responding vs. low responding subjects). These gene clusters were associated in turn with a variety of BTMs corresponding to PBMC functions (Table 1). The Tan cluster, for example, is highly enriched for genes involved in cell cycle and transcription, while the Greenyellow cluster is highly enriched for genes involved in B cell signatures and responses. The Yellow gene cluster contains a large number of genes related to monocytes, cell cycle and transcription, and inflammatory signaling (including innate antiviral responses). The Magenta cluster is highly enriched for genes involved in platelet activation, myeloid cells, and monocytes. The Salmon and Purple clusters are enriched for genes active in NK cells, and the Pink cluster appears to be involved in monocyte and immune activation. Finally, the Green and Black gene clusters are enriched for T cell differentiation and activation. The remaining clusters—Blue, Brown, Cyan, Turquoise, Red, and Grey—were not enriched for any BTM gene signatures with currently known functions.

### Expression of genes in WGCNA-derived gene clusters correlates with different aspects of measured immune responses to vaccination

**Figure 3:**
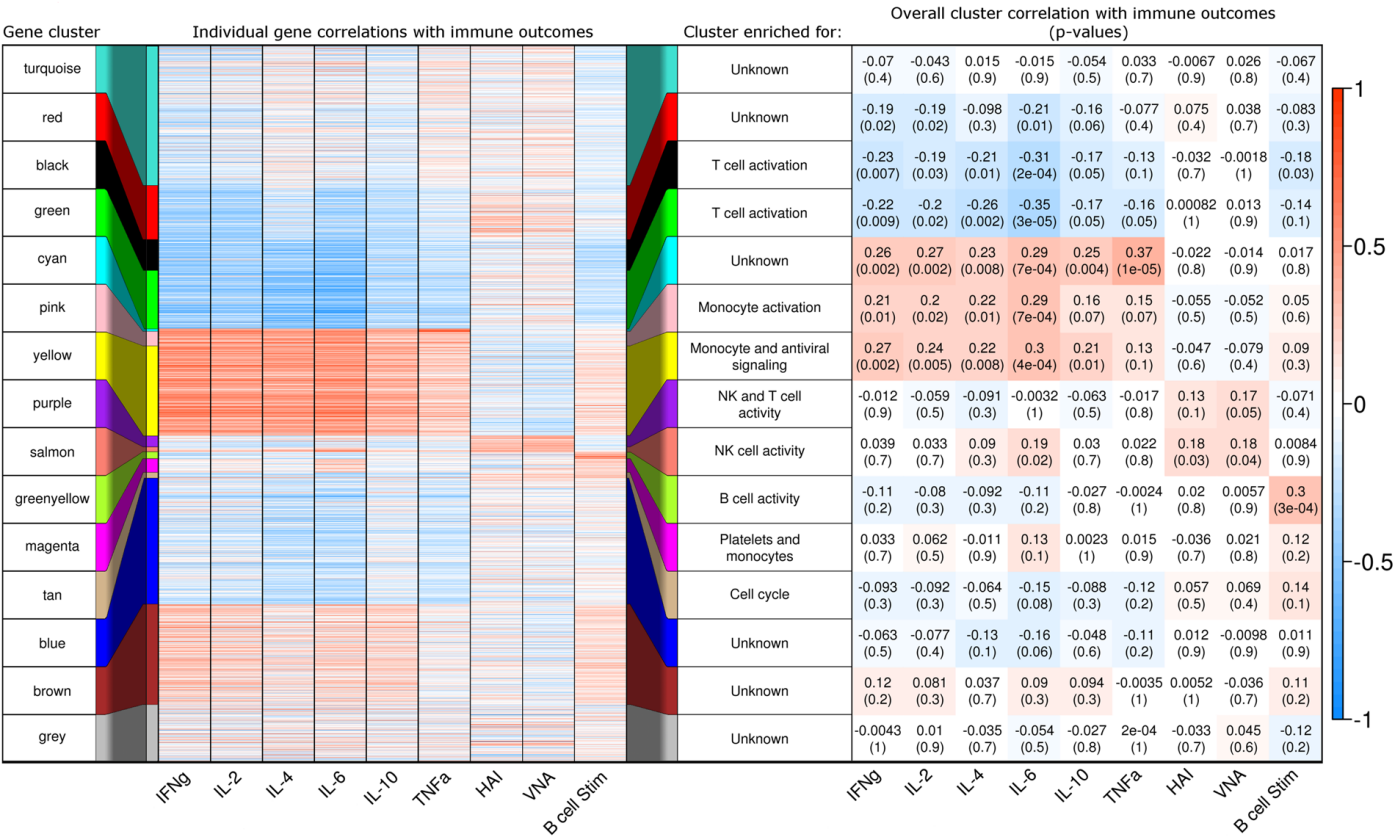
Gene clusters (transcriptomic data 28 days post-vaccination) correlate meaningfully with immune response outcomes after vaccination. Gene expression (mRNA-Seq) data from PBMC samples of 138 older adults after influenza vaccination was clustered using WGCNA. Cluster membership is indicated by color. Left panel: heatmap of the correlation between each gene (row) and subjects’ immune response outcomes – cytokine recall responses (IFN-γ, IL-2, IL-4, IL-6, IL-10, TNF-α), H1N1 antibody responses (HAI/VNA), and memory B-cell responses (B-cell ELISPOT). Center column: cluster enrichment labels from Table 1. Right panel: Pearson’s correlation coefficient and p-value for relationships between cluster eigenvector (representing overall behavior) and subject immune outcomes. Heatmap shading indicates strength of correlations in both panels; see scale bar at right.

To examine the possible function of genes within the WGCNA clusters, we examined the correlation between the expression of each gene and the subjects’ measured immune outcomes: antibody responses (HAI/VNA)^42,44,46^, memory B cell responses (B-cell ELISPOT)^44^, and PBMC cytokine recall responses (secretion of IFN-γ, IL-2, IL-4, IL-6, IL-10, and TNF-α) (Supplementary Table S2). A heatmap of these correlations is shown in Fig. 3, left panel. The eigenvector of each cluster (first principal component of the cluster genes’ transcriptional data, representing the overall expression behavior of genes in the cluster) was also tested for correlation with subjects’ immune outcomes using Pearson’s correlation test (Fig. 3, right panel).

The WGCNA clustering algorithm separated the 14,197-gene transcriptomic dataset into biologically meaningful clusters of similarly behaving genes. The WGCNA-derived gene clusters that were highly enriched for specific immune-related gene functions (Table 1) also exhibited significant correlations with relevant subject immune outcome measures. Gene clusters that were enriched for T cell activation, antiviral signaling, and monocyte activation proved to be correlated with PBMC cytokine recall responses (Red, Black, Green, Cyan, Pink, Yellow; 4,963 total genes). Gene clusters enriched for NK and T cell activity (Salmon and Purple; 321 genes total) were found to correlate with subject antibody responses (HAI and VNA titers).

The cluster most strongly associated with B-cell activity (Greenyellow; 135 genes) was appropriately found to strongly correlate with subject B-cell ELISPOT responses. Thus, through this clustering technique, the total transcriptomic dataset (14,197 genes) was narrowed to 135 similarly acting genes likely involved in the development and maintenance of influenza A/H1N1-specific B cells. Notably, these 135 genes are not significantly correlated with PBMC cytokine recall responses to influenza A stimulation. B cell ELISPOT activity is also more weakly correlated with another gene cluster enriched for T cell activity (Black cluster; 629 genes).

### WGCNA cluster gene membership allows for identification of biologically-relevant gene markers of vaccine-induced immunity

To see if WGCNA clustering of gene expression data can be used to detect biologically relevant gene markers of vaccine-induced immunity, we examined the gene membership in the WGCNA clusters that correlate with subject immune outcomes. The top five genes in each cluster—with behavior most closely mirroring each cluster’s “average” eigengene behavior—are listed in Table 2.

**Table 2:** Top five genes in immune-related WGCNA gene clusters. The five genes in each WGCNA cluster that are most closely correlated with the cluster eigengene are listed, along with their correlation with the relevant immune outcomes.

Immune Outcome	Genes from Cluster:	Cluster enrichment summary	Top 5 genes	Correlation with B cell ELISPOT	Correlation p-value	Gene name
B-cell ELISPOT	Greenyellow	B cell activity	*CD79A*	0.31	2.1E-04	B-cell antigen receptor complex-associated protein alpha chain
			*RALGPS2*	0.30	2.9E-04	Ras-specific guanine nucleotide-releasing factor
			*CD22*	0.28	9.1E-04	B-cell receptor
			*CD19*	0.33	8.1E-05	B-lymphocyte antigen
			*FCRLA*	0.37	7.6E-06	Fc receptor-like A
**Immune Outcome**	**Genes from Cluster:**	**Cluster enrichment summary**	**Top 5 genes**	**Correlation with HAI titers**	**Correlation p-value**	**Gene name**
Antibody titers	Salmon	NK cell activity	*SPON2*	0.24	4.0E-03	Spondin-2
			*KLRF1*	0.19	2.5E-02	Killer cell lectin-like receptor subfamily F member 1
			*AKR1C3*	0.23	6.3E-03	Aldo-keto reductase family 1 member C3
			*NCR1*	0.26	2.4E-03	Natural cytotoxicity triggering receptor 1
			*PRF1*	0.20	1.8E-02	Perforin-1
	Purple	NK and T cell activity	*FCRL6*	0.11	1.9E-01	Fc receptor-like protein 6
			*GZMA*	0.15	8.3E-02	Granzyme A
			*MATK*	0.16	6.6E-02	Megakaryocyte-associated tyrosine-protein kinase
			*CST7*	0.12	1.6E-01	Cystatin-F
			*NKG7*	0.15	7.5E-02	G-CSF-induced gene 1 protein
**Immune Outcome**	**Genes from Cluster:**	**Cluster enrichment summary**	**Top 5 genes**	**Correlation with** ***in vitro*** **IL-6 secretion**	**Correlation p-value**	**Gene name**
PBMC cytokine recall	Yellow	Monocytes and inflammation	*ANKRD50*	0.22	8.2E-03	Ankyrin repeat domain-containing protein 50
			*RAB31*	0.22	1.1E-02	Ras-related protein Rab-31
			*CORO1C*	0.29	6.6E-04	Coronin-1C
			*C8orf83*	0.21	1.4E-02	Triple QxxK/R motif-containing protein
			*CD68*	0.22	9.7E-03	Macrosialin
	Pink	Monocyte activation	*USP32*	0.23	6.2E-03	Nucleolar transcription factor 1
			*VPS37C*	0.26	2.3E-03	Ubiquitin carboxyl-terminal hydrolase 32
			*DIRC2*	0.23	7.9E-03	Vacuolar protein sorting-associated
			*NHS*	0.13	1.2E-01	Disrupted in renal carcinoma protein 2
			*STX11*	0.25	2.8E-03	26S protease regulatory subunit 8
	Cyan	None	*PTPRS*	0.19	2.9E-02	Receptor-type tyrosine-protein phosphatase E
			*KCNK10*	0.11	1.8E-01	Potassium channel subfamily K member 10
			*CLEC4C*	0.24	5.1E-03	C-type lectin domain family 4 member C
			*CUX2*	0.19	2.8E-02	Homeobox protein cut-like 2
			*LILRA4*	0.21	1.4E-02	Leukocyte immunoglobulin-like receptor subfamily A member 4
	Green	T cell activation	*RPL13A*	−0.32	1.0E-04	60S ribosomal protein L13a
			*RPL3*	−0.33	9.5E-05	60S ribosomal protein L3
			*OCIAD2*	−0.30	4.3E-04	OCIA domain-containing protein
			*RPL10*	−0.32	1.1E-04	60S ribosomal protein L10
			*SNRPN*	−0.28	1.1E-03	small nuclear ribonucleoprotein-associated protein N
	Black	T cell activation	*CDR2*	−0.32	1.3E-04	Cerebellar degeneration related protein 2
			*FLT3LG*	−0.34	4.7E-05	Fms-related tyrosine kinase 3 ligand
			*MLLT3*	−0.22	8.1E-03	Protein AF-9
			*CAMK4*	−0.36	1.2E-05	Calcium/calmodulin-dependent protein kinase type IV
			*SFMBT1*	−0.27	1.1E-03	Scm-like with four MBT domains protein 1
	Red	None	*PRPF39*	−0.17	4.7E-02	Pre-mRNA processing factor 39
			*ZNF37BP*	−0.21	1.3E-02	Zinc finger protein 37B, pseudogene
			*TUBGCP6*	−0.17	4.8E-02	Gamma-tubulin complex component 6
			*CUL9*	−0.17	5.2E-02	Cullin-9
			*NEURL4*	−0.12	1.4E-01	Neuralized-like protein 4

We found that the top genes in each cluster typically display clear biological association with the summary class (See Table 1) of that cluster, and logically link to the immune outcomes with which they correlate. Some genes are better correlated with immune outcomes than others, and may therefore be better gene markers of these immune outcomes. WGCNA allows creation of gene signatures of different aspects of vaccine-induced immunity with clear biological meaning, using data-driven methods alone, unbiased by current knowledge.

### WGCNA-derived gene clusters represent distinct but interacting immune processes

**Figure 4:**
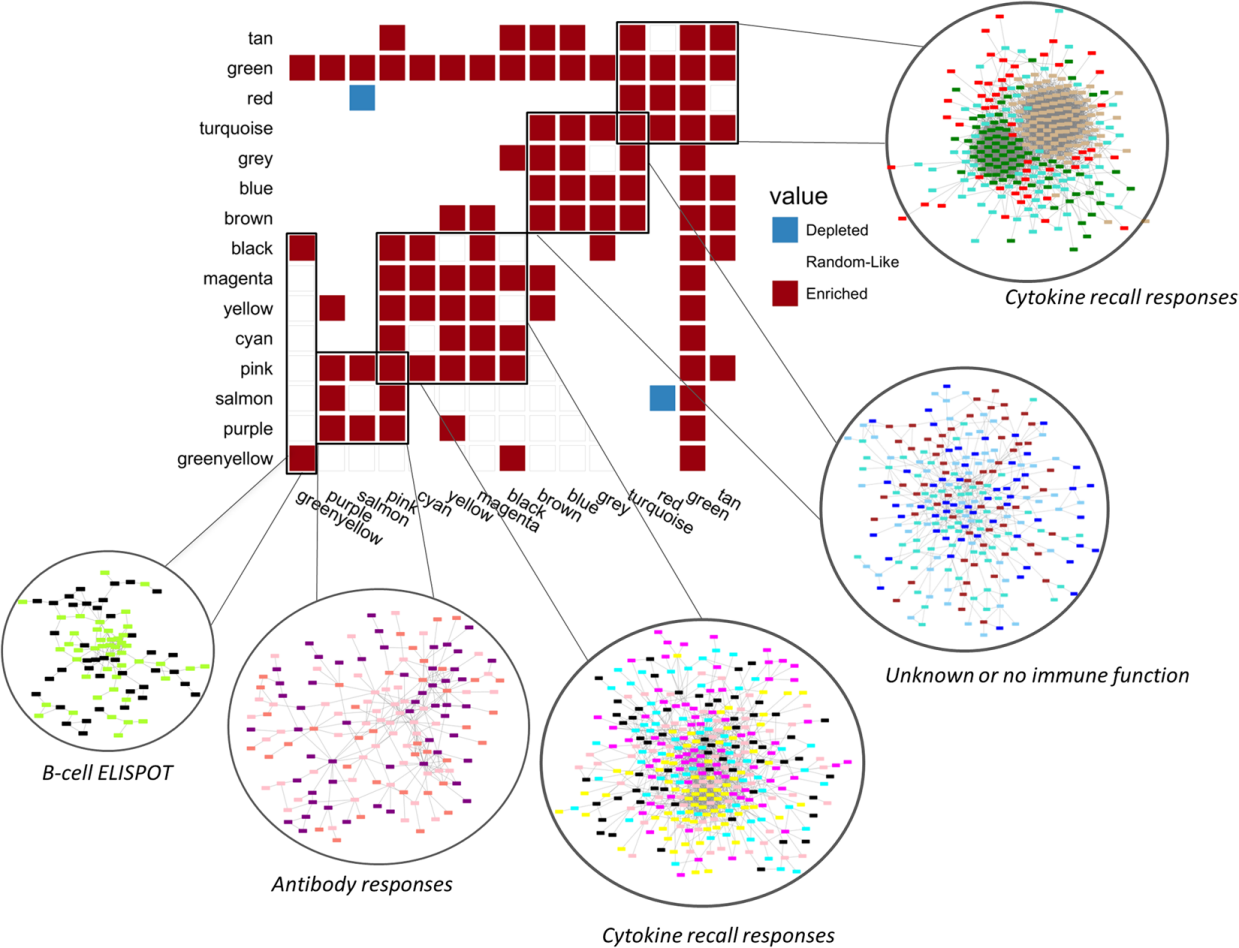
Cluster interconnectivity matrix. The cluster interconnectivity matrix shows gene cluster pairs found to be enriched or depleted for cross-cluster gene interactions. Red squares indicate that two gene clusters are enriched for cross-cluster gene interactions, blue squares indicate two clusters depleted for cross-cluster gene interactions, and a lack of either enrichment or depletion is denoted in white. Groups of highly interconnected gene clusters are identified (black boxes), and network diagrams of these meta-clusters, displaying known gene-gene interactions between the top 100 individual genes from each involved cluster, are displayed in the inset circles.

To characterize the relationships of these non-overlapping WGCNA-derived gene clusters with one another, we examined the interaction of genes in each cluster with the genes in each of the other gene clusters. For each pair of gene clusters, we calculated the fraction of genes in one cluster that have known interactions with genes in the second cluster, and used this to calculate a cluster-cluster interaction score indicating whether the two gene clusters are enriched (or depleted) for interacting genes. The matrix of cluster-cluster interconnectivity scores is displayed in Fig. 4. Groups of highly interacting gene-expression clusters can be seen along the diagonal.

The interconnectivity analysis resulted in five major groups of gene clusters, as illustrated by the boxed clusters in Fig. 4. Highly interacting gene clusters tended to correlate with similar immune response outcomes. For example, the expression of genes in the Yellow, Cyan, and Pink cluster grouping all correlate positively with cytokine responses (Fig. 3). These three clusters represent three sets of genes with different expression patterns from one another, yet interact heavily with one another and correlate with the same immune outcomes. Conversely, the highly interacting Red-Black-Green gene clusters correlate with similar biological responses (negatively correlated with cytokine recall responses from PBMCs), yet the Black cluster is not particularly interconnected with the Red cluster. This illustrates that different genesets may contribute to the same immune outcomes, yet they do not display high levels of gene-gene interactions. The Green cluster is highly interconnected with all other clusters, possibly through the inclusion of a few particularly well-linked genes (i.e., transcription factors, ribosomal proteins).

The Salmon, Purple, and Pink clusters are enriched for interacting genes. The antibody-correlated Salmon and Purple clusters interact with Pink cluster genes, which appear to link these influenza antibody-response related genes with those involved in cytokine recall responses (i.e., the Cyan, Yellow, Magenta, and Black clusters). While the Salmon and Purple cluster genes interact highly with one another, and both of these clusters correlate with antibody titers, we find evidence that they do have distinct behavior from one another: the Salmon cluster is also correlated with IL-6 responses, while the Purple cluster is not. Finally, the Greenyellow cluster interacts solely with the Black cluster, which is the only other gene cluster correlated with B-cell ELISPOT outcomes. This suggests that these genes are exclusively involved in driving ELISPOT responses.

### Time development of correlations between PBMC cytokine recall responses with gene expression clusters reveals signatures of pre-existing immunity

**Figure 5:**
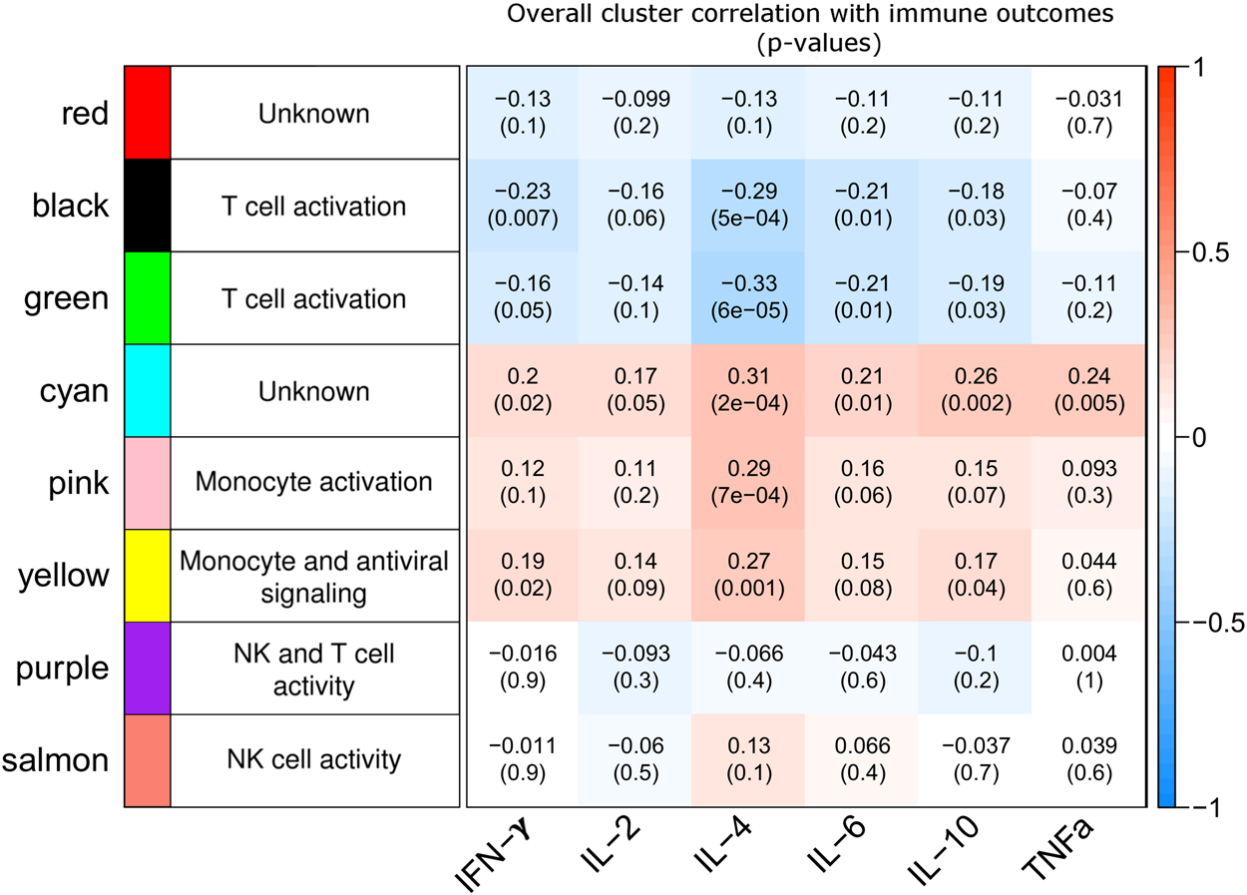
PBMC gene expression *prior* to vaccination (Day 0) correlates with post-vaccination (Day 28) PBMC cytokine recall responses. Gene expression data at baseline (Day 0) was clustered according to the Day 28 WGCNA gene clusters, and the correlation coefficient of each cluster’s Day 0 eigengene with PBMC cytokine responses is shown, colored as in previous figures.

To look for pre-existing gene expression signatures that predict influenza immune outcomes, we examined the relationship of baseline (Day 0) gene expression data with subject immune outcomes 28 days after vaccination. To do so, we clustered Day 0 gene expression data according to WGCNA clusters described above. The correlation of each gene cluster’s eigengene (median expression behavior) at Day 0 with PBMC cytokine recall responses measured at Day 28 is shown in Fig. 5.

Genes in clusters related to PBMC cytokine recall responses showed baseline (Day 0) expression levels that correlated with post-vaccination PMBC cytokine responses. This correlation suggests the presence of significant pre-existing cellular immunity to influenza virus. This method has identified transcriptomic signatures of pre-existing cellular immunity embedded in PBMC transcriptomic profiles prior to vaccination. We found transcriptional signatures of preexisting immunity for PBMC cytokine responses specific to influenza A/H1N1, but did not find equivalents for antibody HAI/VNA or memory B-cell ELISPOT responses to the same virus.

### Time development of correlations between humoral and cellular immunity measures and gene expression clusters reveals stages of humoral and cellular vaccine response

**Figure 6:**
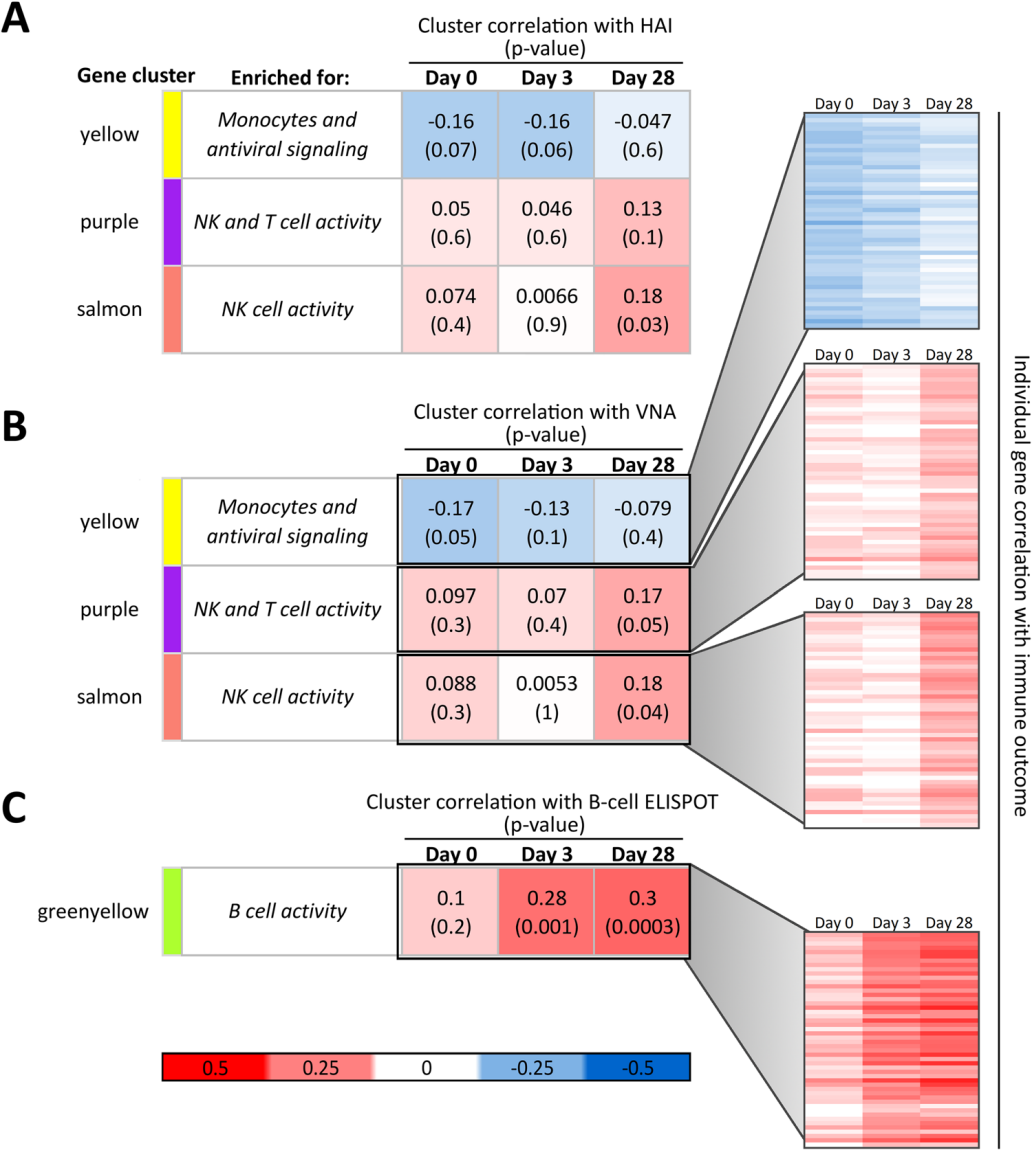
Time development of gene expression correlation with antibody and B-cell ELISPOT immune outcomes reveals temporal patterns leading to vaccine responses. Gene expression data at baseline (Day 0), Day 3, and Day 28 after vaccination was clustered according to the Day 28 WGCNA gene clusters, and the correlation of each cluster’s eigengene with final antibody (panel A) and B-cell ELISPOT (panel B) immune outcomes was calculated at each time point. Inset panels: time development of individual genes’ correlation with indicated immune outcomes for the top 50 genes in each displayed cluster.

To further examine the development of our gene expression correlations with antibody and B-cell ELISPOT, for which we did not find transcriptomic signatures of pre-existing immunity, we clustered baseline and early responding gene expression data (Day 0 and Day 3) according to the WGCNA clusters developed using the Day 28 gene expression data. The correlation of the eigengene (overall behavior) of each gene cluster at each time point with final antibody (VNA) and memory B-cell (ELISPOT) responses was also calculated. Significant correlations are shown in Fig. 6, along with correlations between each of the top 50 genes in each cluster and the corresponding Day 28 immune outcome (inset panels).

Development of the transcriptomic clusters’ correlation with immune outcomes reflects the progression of immune responses to vaccination. Initially (Day 0), the Yellow “monocytes and inflammation” gene cluster shows significant negative correlation with peak antibody (VNA) titers, which disappears after vaccination. Late in response, the Purple “NK and T cell activity,” and Salmon “NK cell activity” gene clusters show correlation with antibody titer (Fig. 6A).

While baseline expression of genes in the Greenyellow gene cluster does not correlate with B-cell ELISPOT responses to vaccination, transcriptomic activity of these genes three days after vaccination is related to ultimate ELISPOT responses, and this relationship is maintained through Day 28 (Fig. 6B). Expression of genes in the Greenyellow cluster appears to be important for development of B-cell immune memory to the influenza vaccine strains, both early and late in responses.

## Discussion

Development of humoral and cellular immunity to influenza after seasonal influenza vaccination is a complex process integrating the activity of multiple immune cells. Transcriptomic data from human PBMCs sampled post-vaccination contains a wealth of valuable information about human immune responses to vaccination; however, such data is complex and difficult to meaningfully analyze. We used WGCNA, a hierarchical clustering technique, to examine PBMC gene expression data from 138 subjects before and after vaccination and more fully examine relationships between gene expression and subject immune outcomes.

When applied to post-vaccination PBMC transcriptomic data, the data-driven WGCNA algorithm clustered the 14,197 genes into 15 gene clusters, which ranged in size from 2,790 to 54 genes. Gene expression in nine of these clusters (5,419 genes) demonstrated significant correlation with immune responses measures. Six gene clusters (4,963 genes) correlated with PBMC cytokine recall responses, and two clusters (321 genes) correlated significantly with influenza A/H1N1 neutralizing antibody measures (Purple, Salmon). A single small 135-gene cluster (Greenyellow) correlated strongly with B-cell ELISPOT measures.

### Gene expression relationships with post-vaccination PBMC cytokine recall responses

Cross-reactive T-cell and other cellular memory responses are important for long-term protection against influenza in older adults. While not yet well worked out, *ex vivo* cellular immune response measures have been associated with vaccine-induced protection in the elderly, while serum antibody response may be a poorer measure of vaccine efficacy in older people when used alone^6,68,69^.

Our data identified gene clusters related to cellular immunity after vaccination, potentially elucidating the processes underlying the development of this immunity. Of the six gene clusters that correlated with our *ex vivo* measures of PBMC cytokine recall responses, we identified two negatively correlated clusters (Black, Green) enriched for T cell genes. The genes whose behavior is most representative of these clusters included ribosomal proteins (RPL13A, RPL3, RPL10, SNRPN), and FLT3L, which is a key gene acting to support dendritic cell action and enhance systemic T cell and humoral immunity after vaccination^70,71^. CAMK4, a kinase previously implicated in transcriptional regulation in lymphocytes^72^, and MLLT3 (aka AF9), a transcriptional regulator known to be involved in hematopoiesis and involved in monocyte-macrophage maturation^73,74^, were both prominent members of the Black gene cluster, suggesting that these genes have significant roles in immune responses to vaccination. CAMK4 has been previously identified as a key negative regulator of antibody responses to trivalent seasonal influenza vaccines^25^, thus validating the ability of WGCNA to identify key genes regulating immune responses after vaccination. The role of MLLT3 in vaccine responses has not been previously described. MLLT3 is a component of the super elongation complex that functions to increase the rate of RNA polymerase II transcription. MLLT3 is a regulator of human hematopoiesis, where mutations are associated with various leukemias^75^, and increased expression promotes the output of erythroid and megakaryocytic progenitors^73^. It is possible that lower expression of this protein instead encourages the formation of monocytes, thus affecting immune responses to vaccination. Such hypotheses should be further explored in future studies.

We also detected transcriptomic signatures of pre-existing cellular immunity to influenza (Day 0 gene expression that correlated with PBMC cytokine secretion) in these Red, Black, Green, Cyan, Pink, and Yellow clusters that correlated with cytokine recall responses. We note that no equivalent transcriptional signatures for pre-existing antibody or B-cell ELISPOT responses were found. This may indicate that pre-existing B cell responses require little maintenance on a transcriptional level, and/or may reflect that influenza-specific memory B cells reside predominantly outside of the blood and their transcriptional signatures may be too weak to detect in PBMCs.

Interestingly, when the WGCNA algorithm was applied to fold-change gene expression datasets, the resulting gene clusters were larger in size, fewer in number, and did not correspond to biological functions (via enrichment analysis). We believe this is due to pre-existing immunity, as seen in Fig. 5. For subjects with high levels of pre-existing immunity, subtracting the baseline gene expression data, which already captures this pre-existing immunity, from the Day 28 post-vaccination responses (once again in immune homeostasis) may obscure the existing signature of immunity.

Accounting for pre-existing immunity to influenza virus is a considerable challenge in studies of influenza vaccine-induced immune responses, yet maintenance of cellular immunity via annual revaccination is important for protection in older adults^76,77^. Our study provides insight into the genes involved in this pre-existing cellular immunity.

### Gene expression relationships with humoral immune responses

Baseline gene expression of the Yellow gene cluster enriched for genes involved in monocyte induction, cell cycle & transcription, and inflammatory/antiviral signaling was found to be negatively correlated with antibody titers. This correlation disappears post-vaccination. Curiously, in addition to genes related to monocytes and inflammation, the Yellow gene cluster includes multiple toll-like receptors (TLRs) known to be involved in innate responses (*TLR1*, *4*, *5*, *6*, *7*, and 8) that have been previously identified as part of early (Day 3) gene signatures *positively* predicting antibody responses to seasonal influenza vaccine^25,51^. This finding is consistent with findings of Nakaya *et al*.^23^: in a meta-analysis of multiple influenza vaccine studies, the group found baseline expression in monocyte- and cell cycle-related gene modules that included many TLRs (BTMs M11.0 and M4.0, corresponding to many genes in our Yellow cluster) that also correlate negatively with antibody titers^23^. Our findings lend further support to the hypothesis that baseline inflammation may inhibit the induction of vaccine-induced antibody responses, as proposed by others^23,78,79^. Interestingly, in a comparison of baseline expression in young vs. old subjects, Nakaya *et al*.^23^ also found increased levels of monocyte cell populations and corresponding monocyte-related gene expression, suggesting that monocyte activity may be a key mediator of inflammation and a correspondingly reduced ability of the elderly to develop antibody responses to influenza vaccination.

Of the five genes whose behavior most strongly mirrored that of the Yellow gene cluster as a whole, two—*CORO1C* and *C8orf83*—are less well-studied and have not been previously associated with influenza vaccine responses. *CORO1C* encodes the coronin-1C protein, a member of the WD repeat protein family that is involved in a variety of cellular processes, including signal transduction and gene regulation, and may be involved in fibroblast migration^80^. *C8orf83* encodes TRIQK, which is a small protein highly conserved across vertebrates with largely unknown function and may have undiscovered roles in immunity^81^. These genes may play heretofore undescribed roles in the development of immunity in response to influenza vaccine.

Day 28 gene expression of two WGCNA gene clusters (Purple and Salmon; 321 total genes) correlated significantly with HAI and VNA antibody measures; however, in these clusters, gene expression in Days 0 and 3 demonstrated no correlation with antibody titers. The genes in these clusters were found to be enriched for genes involved in the control of NK and cytotoxic T cells rather than B cells. While NK and dendritic cells are involved early in the induction of immune responses leading to antibody secretion^82^, the fact that no correlation was observed between baseline/Day 3 expression of these NK-related genes and antibody measures leads us to believe that the genes in the Purple and Salmon gene clusters may not be directly involved in the *induction* of antibody responses, but rather in processes that parallel the development of antibody-secreting B cells. In the Purple and Salmon NK-related gene clusters, eight and four genes, respectively, are KIR and KLR (killer cell immunoglobulin-like and lectin-like receptor) genes, which are known to be inhibitory receptors expressed by NK and CD8^+^ T cells and inhibit their cytotoxic activity^83^. This leads us to believe that higher expression in these clusters may lead to lower cytotoxic activity, subsequent lower levels of inflammation, and culminate in high levels of antibody production. Others have noted that the frequency, type, and activity of NK cells tends to change with age^23,24,84–86^, with increased frequencies of NK and CD8^+^ T cells, and corresponding gene expression changes. Thus, these Purple and Salmon gene clusters may serve as post-vaccination indicators of successful humoral responses in older populations, and may also account for immunosenescence.

Additional genes found in the Salmon clusters of note include *SPON2 and AKR1C3* genes. *AKR1C3* encodes a protein involved in sex steroid metabolism^87^. Expression of *AKR1C3* is regulated by IL-6^88^, appears to change in PBMCs during aging, and may be an endocrine link with immunosenescence^89^. Further investigation of this gene may help us better understand the poor responses of the elderly to influenza vaccine. The *SPON2* gene encodes Spondin-2, also known as mindin. Mindin is an essential component of the innate immune response, as it is a pattern recognition molecule that binds to macrophage-presented receptors during responses to pathogens^90^. Mindin has previously been associated with the intranasal clearance of influenza viruses in mice^91^, by both binding directly to influenza virus particles and also activating macrophages. Mice lacking mindin have been demonstrated to have impaired responses to bacterial and influenza virus infections^90,91^, indicating a potentially critical role in influenza vaccine responses that has not previously been reported and should be further studied.

Additional prominent genes in the Purple gene cluster include *MATK* (encoding megakaryocyte-associated tyrosine-protein kinase), which is an essential regulator of immune cells in mice^92^. MATK may play a role in signal transduction during megakaryocytopoiesis and may enhance MAPK activation in RAS-mediated signaling^93^. *CST7* encodes cystatin-F, an immune cell-specific inhibitor that plays a role in immune responses through regulation of specific enzyme targets such as cathepsin C, which in turn regulate the cytotoxicity of NK and T cells^94–96^. Thus, these two genes not previously connected with influenza vaccine responses may well influence immune responses to influenza vaccine and are candidates for future studies.

B-cell ELISPOT measures correlated strongly with one small gene cluster (Greenyellow, 135 genes) whose Day 0, Day 3, and Day 28 expression patterns were found to be largely unassociated with antibody titers. The Greenyellow cluster is dominated by B-cell genes, with four of the top five genes involved in the B-cell antigen receptor complex (CD19, CD22, CD79A, FCRLA). The final top gene, RALGPS2, is a RAS-specific GTPase implicated in cell survival and cell cycle control^97^. Interestingly this gene’s expression patterns grouped it with B-cell related genes, rather than with other cell cycle-related genes in the Brown or Yellow gene clusters. This suggests a potential role of RALGPS2 in B-cell activity beyond that of other RAS-specific GTPases.

Many of the B cell-related genes found in our Greenyellow cluster were found by others^25^ to correlate with antibody responses; however, these correlations were measured at Day 7 post-vaccination and reflect peak antibody-secreting cell (ASC) populations and activity^24^, a timepoint our study did not include. If a future study were conducted with a Day 7 timepoint, we would expect to find such correlations between Day 7 ASC gene expression and antibody titer.

A previous study examining a subset of this dataset used per-gene logistic regression models to identify individual genes whose expression correlated with HAI response across 94 subjects^51^. Of the 30 top-associated genes in that study, nine are members of WGCNA gene clusters associated with immunity, of which just six are found in our HAI-associated Salmon or Purple clusters. Individual genes from this transcriptomic dataset that correlated with B-cell ELISPOT responses were also previously identified^64^. Notably, just a single gene from this previous study, *USP6NL*, was clustered into our B-cell ELISPOT-associated Greenyellow cluster. These results indicate that logistic regression models and WGCNA analysis highlight significantly different genes and genesets. Langfelder *et al*. demonstrated that, in biological systems where marginal associations are weak or noisy, WGCNA gene cluster membership is more reproducible than single-gene selection by marginal^37^. Thus, the use of WGCNA as an alternative or additional method for analysis of large-scale post-vaccination gene expression data may prove invaluable for future studies. Additionally, WGCNA identification of genes whose expression follows patterns similar to other immune-relevant genes may allow for discovery of genes with important but previously undescribed immunological roles.

### Strengths and limitations of the study

The transcriptome of human PBMCs after influenza vaccination reflects contributions from many cell subsets and cellular processes leading to protective immunity, yet such data is highly complex and difficult to interpret. Studying individual immune cell subsets is a common strategy to simplify gene expression datasets; however, this reductionist approach leaves much to be desired. While transcriptomic profiles of each cell subset differ significantly from the profiles of PBMCs^28^, measurement of gene expression in all possible or even all relevant cell subsets is a difficult and costly process. Additionally, as immune cell subsets signal to and affect one another, reductionist study of individual cell subsets necessarily disrupts or ignores the behavior of the immune system as a whole. The WGCNA algorithm appears to be capable of separating genes involved in different T- and B-cell functions (i.e., cytokine secretion vs. cytotoxic responses, and cytokine-producing vs. antibody-producing, respectively) from whole-PBMC transcriptomic data. We propose the use of WGCNA as a potential tool with which to study transcriptomic responses after vaccination without the disadvantages of a reductionist approach.

Multiple hypothesis testing is always a consideration in high-dimensional studies. The Bonferroni correction, which assumes independence, is inappropriately strict in systems with strong correlations between statistical tests. We present the actual p-values to allow the reader to draw their own conclusions about the significance of the data, as others in the field have also done. Future validation work will be conducted to confirm our results.

Use of a trivalent seasonal influenza vaccine that contains influenza A/H1N1, influenza A/H3N2, and influenza B is a study limitation. While our gene expression data (mRNA-seq) reflects responses to vaccination with all three influenza strains, due to practical limitations we conducted studies of immune development (PBMC cytokine recall responses after *ex* vivo influenza stimulation, B-cell ELISPOT, antibody assays) specific to just influenza A/H1N1. Despite this, relationships between gene expression and immune outcomes are expected, as the development of immunity to the three virus strains occurs concurrently. Further studies may now be conducted on stored PBMC samples to examine the development of cellular and humoral immunity to the other two influenza strains.

## Conclusions

Through WGCNA clustering of PBMC gene expression data collected after trivalent seasonal influenza vaccination, we identified individual gene clusters whose expression correlated with subject cellular, memory B-cell and antibody responses. This clustering procedure allowed us to identify key marker genes that reflect development of vaccine-induced immunity. Our findings validated gene signatures of influenza A/H1N1 vaccination responses previously identified by others, and expanded upon these by identification of previously uncharacterized proteins with possible immunological roles. Further analysis suggested the involvement of different genes and immune cell subsets at key points in the development of long-term immunity after influenza virus vaccination. These results may help elucidate the roles that genes and immune cell subsets play during human immune responses to seasonal influenza vaccination, and suggest further utility of the WGCNA gene clustering algorithm in vaccinology.

Further work may use WGCNA clustering to examine the effects of subject characteristics such as age, sex, and obesity on pre- and post-vaccination gene expression, and the relationship of these differentially expressed genes with vaccine-induced immunity. Additional, future studies may examine the behavior of the cell subsets identified in this report as likely contributors to particular aspects of vaccine-induced immunity to influenza, allowing us to identify the mechanisms by which these cells contribute to immune response. This information may be used in the future to reverse-engineering novel influenza vaccine candidates that trigger directed immune responses to enhance development of protection.

## Electronic supplementary material

Supplementary Information

S1 Table

S2 Table
