# Supplementary material for: Transcriptomic signatures of cellular and humoral immune responses in older adults after seasonal influenza vaccination identified by data-driven clustering
Source: Sci Rep. 2018 Jan 15;8:739. doi: 10.1038/s41598-017-17735-x (PMC5768803; doi:10.1038/s41598-017-17735-x)
Supplement: Supplementary file 1 — Supplementary Information [file 41598_2017_17735_MOESM1_ESM.pdf]

Transcriptomic signatures of cellular and humoral immune responses in older adults after seasonal influenza vaccination identified by data-driven clustering

Emily A. Voigt[1], *Diane E. Grill*[2], *Michael T. Zimmermann*[2], *Whitney L. Simon*[1],

Inna G. Ovsyannikova[1], *Richard B. Kennedy*[1], *Gregory A. Poland*[1,]*

[1]Mayo Clinic Vaccine Research Group,

[2]Division of Biomedical Statistics and Informatics

Mayo Clinic, Rochester, MN 55905 USA

*Corresponding Author

Short Title: Signatures of influenza vaccine responses in older adults.

Keywords: Influenza Vaccines; Cytokines; Antibodies; Immunity; Immunity, Humoral; Immunity, Cellular; Aging; Gene Regulatory Networks; WGCNA

Abstract Word Count: 200

Total Word Count (excl. Abstract): 6,568

Word Count (excl. Abstract, Methods, References, Figure Legends): 6,568

Address correspondence to:

Gregory A. Poland, M.D., Director, Mayo Vaccine Research Group, Mayo Clinic, Guggenheim 611C, 200 First Street SW, Rochester, Minnesota 55905

Phone: (507) 284-4968; Fax: (507) 266-4716; Email: poland.gregory@mayo.edu

©Copyright 2017 Mayo Foundation for Medical Education and Research

Supplementary Table Legends

S1 Table: WGCNA gene cluster membership. All 14,197 genes from the transcriptomic analysis are listed, along with their corresponding WGCNA gene cluster, cluster membership value, and cluster membership p-value.

S2 Table: Cluster top genes' correlation with immune outcomes. The top 100 genes in each WGCNA gene cluster whose expression best correlates with the cluster eigengene are listed, along with correlations of each gene with major immune outcomes (cytokine secretion, HAI/VNA antibody measures, B-cell ELISPOT measures).
